# *Aedes* (*Georgecraigius*) *epactius* from Zacatecas and Chihuahua Mexico: New Geographical Distribution and Altitude Records

**DOI:** 10.3390/insects15110833

**Published:** 2024-10-24

**Authors:** Valeria H. Ramos-Lagunes, S. Viridiana Laredo-Tiscareño, Rodolfo González-Peña, Jaime R. Adame-Gallegos, Carlos A. Rodríguez-Alarcón, Erick de Jesús de Luna-Santillana, Luis M. Hernández-Triana, Lucia E. Velasco-Chino, A. Gabriela Laredo-Tiscareño, Javier A. Garza-Hernández

**Affiliations:** 1 Instituto de Ciencias Biomédicas, Universidad Autónoma de Ciudad Juárez, Ciudad Juárez 32310, Chihuahua, Mexico; al205078@alumnos.uacj.mx (V.H.R.-L.); viridiana.laredo@gmail.com (S.V.L.-T.); carrodri@uacj.mx (C.A.R.-A.); al208645@alumnos.uacj.mx (L.E.V.-C.); 2 Laboratorio de Arbovirología, Centro de Investigaciones Regionales “Dr. Hideyo Noguchi”, Universidad Autónoma de Yucatán, Mérida 97225, Mexico; fitho.gleez@gmail.com; 3 Facultad de Ciencias Químicas, Universidad Autónoma de Chihuahua, Chihuahua 31125, Mexico; jadame@uach.mx; 4 Laboratorio Medicina de la Conservación, Centro de Biotecnología Genómica del Instituto Politécnico Nacional, Reynosa 88710, Tamaulipas, Mexico; edeluna@ipn.mx; 5 Rabies and Wildlife Zoonoses Research Group, Virology Department, Animal and Plant Health Agency, Addlestone KT15 3NB, UK; luis.hernandez-triana@apha.gov.uk; 6 Department of Nutrition, Campus Fresnillo, Universidad Autónoma de Durango, Fresnillo 99000, Zacatecas, Mexico; laredo.ana.gabi@gmail.com

**Keywords:** *Aedes epactius*, new record, high altitude, Zacatecas, Chihuahua, Mexico, DNA COX1 barcode

## Abstract

Adults and immatures of *Aedes epactius* were collected in July and December 2022 at sites of high elevation in the states of Chihuahua (2300 masl) and Zacatecas (2182 and 2595 masl), Mexico, respectively. Mosquitoes were identified morphologically and sequenced for a DNA barcode of the cytochrome c oxidase I (COX1). This is the first distributional record of *Ae. epactius* in Zacatecas and provides evidence of the highest altitude in the Americas, including Mexico. The geographical distribution of *Ae. epactius* in Mexico was reviewed, and the COX1 analysis, using phylogenetic Bayesian analysis to confirm species identification, was performed.

## Simple Summary

In this work, we report a new distribution for the Western Rock Pool mosquito, *Aedes epactius*, in the state of Zacatecas, Mexico. Additionally, we document its occurrence at 2300 m above sea level in the state of Chihuahua and at nearly 2600 m above sea level in Zacatecas. These records highlight the remarkable dispersal plasticity of the Western Rock Pool mosquito in the Americas, demonstrating its ability to adapt to a wide range of altitudes, from coastal areas with warm temperatures to high-altitude regions with considerably lower average temperatures.

## 1. Introduction

The mosquito *Aedes* (*Georgecraigius*) *epactius* Dyar & Knab, commonly known as the Western Rock Pool mosquito, has not been conclusively incriminated as an arbovirus vector [1]. However, laboratory studies have demonstrated its potential as a competent vector for the Jamestown Canyon virus [2] and its ability to vertically transmit the St. Louis encephalitis virus [3]. Zuñiga et al. (2023) [4] documented WNV-RNA-positive *Ae. epactius* mosquitoes in the municipalities of Casas Grandes (1488 masl), Nuevo Casas Grandes (1460 masl), Praxedis G. Guerrero (1085 masl), Guadalupe (1093 masl), and Ciudad Juárez (1230 masl), in the state of Chihuahua, northwest Mexico. Previously, Correa-Morales et al. (2019) [5] found *Ae. epactius* mosquitoes in both urban and semi-urban areas of Mexico, with some pools testing positive for Zika virus (ZIKV) RNA. In the Americas, the geographic distribution of *Ae. epactius* is widespread, with records in Costa Rica, El Salvador, Guatemala, Honduras, Panama, and the U.S. [5,6,7]. In Mexico, extensive studies on mosquito fauna have been documented, as well as their distribution across multiple regions, including the states of northwest Mexico, the Gulf of Mexico region, the South Pacific, and the Neovolcanic Axis [8,9,10]. Notably, *Ae. epactius* exhibits high plasticity, allowing it to tolerate cold climates at high altitudes [8,9,10,11,12]. The highest previously published elevation record for *Ae. epactius* in Mexico was 2417 masl, where mosquitoes were collected across various elevations and climates in the states of Veracruz and Puebla [9]. These studies underscore the importance of accurately estimating the spatial distribution of *Ae. epactius* and registering precise morphological identification in both immature and adult stages during surveillance operations targeting *Aedes aegypti* and *Ae. albopictus* in Mexico [10,11]. Given the importance of routine surveillance for other Culicidae species incriminated as vectors in Mexico, this research communication reports two significant findings: (1) a new altitude record for *Ae. epactius* in the Americas, including Mexico; and (2) a new distribution record for this species in Zacatecas state, Mexico.

## 2. Materials and Methods

This study is part of a broader investigation into the surveillance of arboviruses in dipteran species of medical and veterinary importance in Mexico. In July and December 2022, resting adults and immature stages of mosquitoes were collected in the states of Chihuahua and Zacatecas. Adults were caught using a buccal aspirator, while immatures were collected using a 50 mL plastic pipette. Immatures were kept in Whirl-pak^®^ plastic bags (Nasco Co., Fort Atkinson, WI, USA) and were reared in individual plastic tubes until imago emergence. Resting adults and recently emerged imagos were killed using chloroform vapors, dried and preserved in sterile 1.5 mL polypropylene tubes, and then kept at −20 °C until processing for morphological identification and molecular analysis. Mosquitoes were identified using an Olympus SZ61 stereomicroscope (Olympus, Tokyo, Japan), and the identification keys of Clark-Gil and Darsie (1983) [13] and Darsie and Ward (2005) [14] were used. Photographs of the key characters were taken using an Olympus SZ61 stereomicroscope with a 55MPixel 4K microscope camera.

For molecular analysis, mosquitoes from each collection site were processed for DNA barcoding and phylogenetic reconstruction to confirm their identification. Briefly, the mitochondrial 5′ cytochrome oxidase region (COX1) was sequenced using the established protocols of Hernández-Triana, L.M., et al. [15]. Overall, COX1 sequences were matched in GenBank using nucleotide BLAST. Then, at least one COX1 reference sequence of some representative species of the subgenera *Aedimorphus*, *Aztecaedes*, *Georgecraigius*, *Howardina*, *Jarnellius*, *Kompia*, *Lewnielsenius*, and *Ochlerotatus*, belonging to the genus *Aedes*, was downloaded from GenBank. Overall sequences were aligned using MUSCLE algorithms and were then compared with our sequences from Chihuahua and Zacatecas by a Bayesian phylogenetic reconstruction analysis. The phylogenetic tree was inferred using the Bayesian algorithm. Sequences were modeled by the general time-reversible (GTR) substitution model, along with Gamma + Invariant sites as the heterogeneity model, using BEAUti software v10.5.0. Then, the tree priors shared by all tree models was computed by the Yule speciation model using a random starting tree, and the MCMC chain was run for 10^8^ generations to ensure convergence. Subsequently, the BEAST file was processed to build the phylogenetic tree. The tree was built using the TreeAnnotator software (v1.10) in the BEAST package with a Burnin (as states) parameter of 10^5^. The tree generated was edited using the FigTree program (v1.4.4) in the BEAST package. An outgroup of the *Lutzomyiz crusiata* COX1 gene was used as the root tree. Finally, an intraspecific genetic distance analysis using the nucleotide substitution model Kimura 2-parameter (K2P) was performed between the sequences of *Ae. epactius* from Chihuahua and Zacatecas and compared with GenBank reference sequences of *Ae. epactius* reported in Mexico, with the aim of verifying low-genetic variability and corroborating that they belong to the same species.

## 3. Results

**Figure 1:**
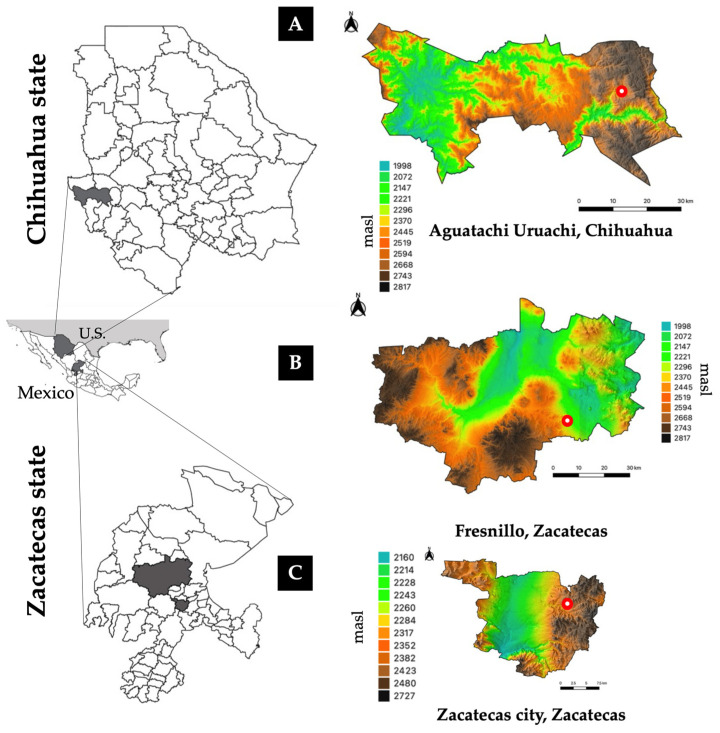
Orographic map of the states of Chihuahua and Zacatecas, showing the municipalities where *Ae. epactius* was found at high altitudes. (**A**) Aguatachi community at 2280 masl, located in the municipality of Uruachi, Chihuahua. (**B**) Zacatecas city in the state of Zacatecas at 2595 masl. (**C**) Fresnillo city at 2182 masl, located in the municipality of Fresnillo, Zacatecas. Red circles with white midpoints indicate the exact site where *Ae. epactius* were collected. The vertical color scales represent elevation in meters above sea level.

*Ae. epactius* was found at high-altitude sites in Aguatachi, a municipality of Uruachi, in the state of Chihuahua (27°54′37″ N; 108°10′14″ W), at 2300 masl. *Ae. epactius* was also found in Zacatecas city (22°46′41″ N; 102°33′46″ W), in a municipality of Zacatecas at 2595 masl, and in Fresnillo city, municipality of Fresnillo (23°10′53″ N; 102°51′46″ W) at 2182 masl, both in the state of Zacatecas (Figure 1). According to the Institute of Statistics and Geography of Mexico (INEGI, in Spanish, 2024) [16], Uruachi is a municipality of Chihuahua, located in the Sierra Madre Occidental (SMO), with an average altitude of 2639 masl, reaching a maximum of 2900 masl, and a minimum of 200 masl; the climate is temperate–subhumid, with average temperatures ranging from 14 °C to 16 °C. The cities of Zacatecas and Fresnillo are situated in a region where the SMO and the Sierra Madre Oriental intersect, resulting in one of the highest elevations in Mexico. In Zacatecas municipality, the average altitude is 2256 masl and ranges from 2800 to 2100 m. In Fresnillo municipality, the average altitude is 2121 masl, with a maximum of 2900 m and a minimum of 1900 m. In both municipalities, the climate is predominantly temperate, ranging from 10 °C to 19 °C.

**Figure 2:**
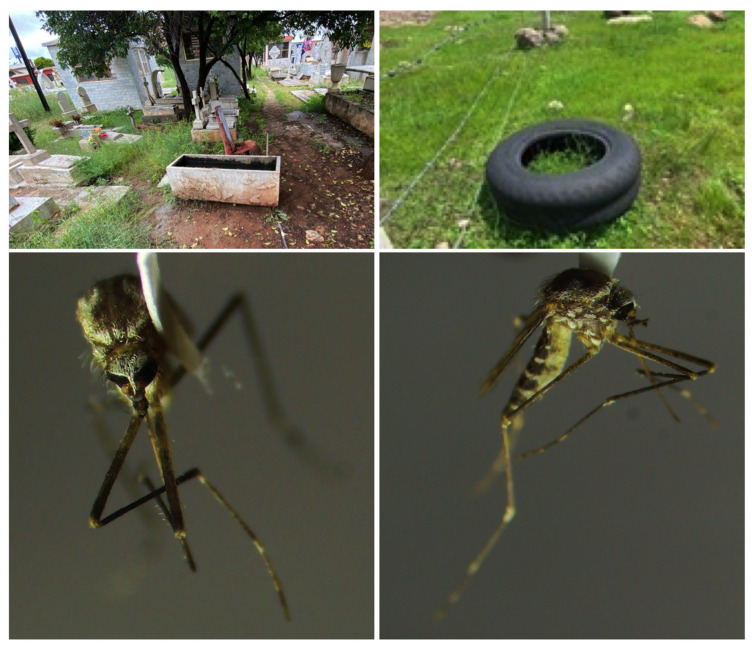
Breeding sites where *Ae. epactius* immatures were found (**top left**). A water tank located in the municipal cemetery in Fresnillo City, Zacatecas (**top right**). A tire roadside in the mountainous region of Urique, Chihuahua. Images of the *Ae. epactius* adults collected in a public restroom in Zacatecas, in the state of Zacatecas, are not included as it was not possible to capture a photograph. (**above left**) *Ae. epactius* female scutellum view. (**above right**) *Ae. epactius* female lateral view.

In the Aguatachi community, seven imagos were obtained from 10 to 15 larvae collected from a tire on the roadside. In Zacatecas City, three adults were found resting indoors in a public restroom. Despite our efforts to locate the breeding site and collect larvae for characterization, we were unsuccessful. In Fresnillo city, 14 imagos were obtained from at least 20 larvae found in a water tank located in the municipal cemetery. Figure 2 shows the *Ae. epactius* female adult morphology and the breeding sites where their immatures were found.

**Figure 3:**
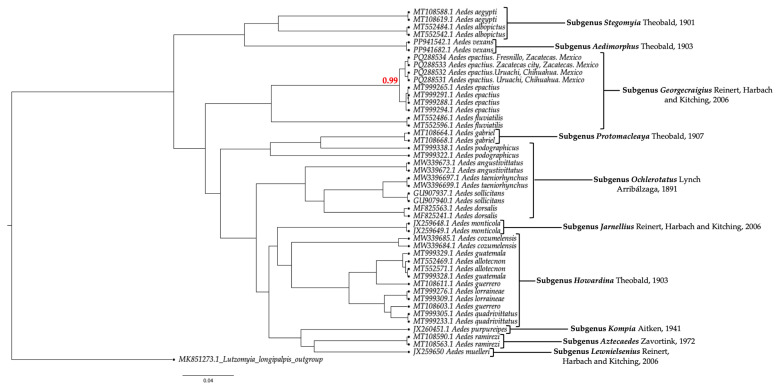
Phylogenetic tree of genus *Aedes* Meigen, 1818. This Bayesian inference tree of COX1 gene nucleotide sequences shows the group of the subgenus *Georgecraigius*, where *Ae. epactius* from Chihuahua and Zacatecas is clustered. The tree is rooted with *Lutzomyia cruciata* as the outgroup. The red number at the *Ae. epactius* node represents posterior probability values, indicating the highly significant probability of the clade formed. The nomenclatural classification of the family Culicidae in this phylogenetic tree is referenced from Wilkerson et al. (2015) [17] and Ortega-Morales et al. (2023) [7].

The COX1 sequences obtained were deposited in GenBank (accession numbers: Urique, Chihuahua: PQ288531 and PQ288532; Fresnillo city, Zacatecas: PQ288533; and Zacatecas city, Zacatecas: PQ288534). Overall, the COX1 sequences from Chihuahua and Zacatecas were compared with those in GenBank using nucleotide BLAST, confirming a strong match with *Ae. Epactius*, with 98% query coverage and 94.75–99.85% identity. The estimation of intraspecific genetic distances between *Ae. epactius* from Chihuahua and Zacatecas, with those from other regions of Mexico, was 0.018 ± 0.001. Phylogenetic analysis confirmed that the genetic relationships of the sequences from *Ae. epactius* in Chihuahua and Zacatecas, when compared with reference sequences from GenBank, were grouped distinctly within the *Ae. epactius* species cluster (Figure 3).

## 4. Discussion

The present communication research supplements existing knowledge on the altitudinal distribution of *Ae. epactius* in Mexico, previously documented by Lozano-Fuentes et al. (2014) [9]. It also provides the first record of the Mexican endemic mosquito species *Ae. epactius* in the state of Zacatecas, Mexico, where it was found at an elevation of 2595 masl, recording the highest altitude at which *Ae. epactius* has been recorded in the Americas, including Mexico, to date.

Lozano-Fuentes et al. (2014) [9] conducted a systematic investigation of *Ae. epactius* across a broad altitudinal and climatic gradient, ranging from near-sea level in Veracruz to high elevations across Veracruz to Puebla, Mexico. The highest altitude where *Ae. epactius* was recorded in Puebla city (19°02′24″ N; 98°11′31″ W), at 2133 masl, where 530 specimens were collected from diverse breeding sites, including discarded containers such as bottles, cans, and plastic bags, as well as larger items like washing machines, refrigerators, tires, buckets, flowerpots, cement troughs, large clay jars, and drums. These records indicate the distribution of *Ae. epactius* at high altitudes in the template–cold climates in Mexico, highlight the species’ remarkable ability to adapt to extreme environmental conditions.

*Ae. epactius* is a species that competes within its niche with other mosquito species. For example, the documented coexistence of *Ae. epactius* with *Ae. aegypti*, an invasive species from Africa, suggests potential competition for ecological niches in similar habitats during their immature stages [11,12]. Additionally, fluctuations in the population dynamics of both species have been observed in response to seasonal changes. For instance, in September—a month characterized by variable temperatures ranging from warm to cold—*Ae. epactius* demonstrates higher densities compared to *Ae. aegypti*. This adaptation to high elevations and cold–temperate climates may have significant implications for vector ecology and the epidemiology of mosquito-borne diseases, influencing mosquito community structures, habitat competition for abiotic resources, predation dynamics, species distribution, and disease transmission [18]. Furthermore, this study presents a new record of the geographic distribution of *Ae. epactius* in Mexico, including Zacatecas, among the states where this species has been documented. Although *Ae. epactius* is not recognized as a major vector of arboviruses, its presence in Zacatecas could have important epidemiological implications. Specifically, *Ae. epactius* may play a ‘protective role’ by potentially limiting the spread of invasive species such as *Ae. aegypti* or/and *Ae. albopictus* [18].

Finally, DNA barcoding based on the analysis of the COI gene is an important tool for species identification and is widely regarded as a key component of integrative taxonomy. It utilizes genetic information to complement and enhance traditional morphological data, improving the accuracy and resolution of species identification [19]. The *Ae. epactius* sequences obtained from Chihuahua and Zacatecas fit precisely with reference sequences within the clade of the subgenus *Georgecraigius* (Figure 3). The calculated low intrageneric distance (less than 2%) indicates that mosquito species in these regions are distributed relatively uniformly, without forming complex clusters, and that the genetic variability within the population is consistent [20].

## 5. Conclusions

This communication research presents the first confirmed distributional record of *Ae. epactius* in Zacatecas and establishes a new highest altitude record for this species in the Americas, including Mexico, suggesting the importance of vector surveillance in regions of high altitude in Mexico. From a wider perspective, generating additional COI gene sequences from various biogeographic regions of Mexico would be of great importance for studying the biology of *Ae. epactius*. This would enable more robust biogeographic analyses and help determine whether multiple lineages of *Ae. epactius* exist in Mexico. Such research is crucial, as a deeper understanding of *Ae. epactius*’ genetic variability could uncover evolutionary patterns, local adaptations, and potential ecological and behavioral differences, all of which could contribute to more effective control and management strategies for this species.

## Data Availability

The original contributions presented in the study are included in the article, further inquiries can be directed to the corresponding authors.
